# Determinants of child wasting in Bhutan. Insights from nationally representative data

**DOI:** 10.1017/S1368980016002111

**Published:** 2016-08-30

**Authors:** Víctor M Aguayo, Nina Badgaiyan, Laigden Dzed

**Affiliations:** 1 UNICEF, Regional Office for South Asia, PO Box 5815, Lekhnath Marg, Kathmandu, Nepal; 2 Nutrition Programme, Ministry of Health, Royal Government of Bhutan, Thimphu, Bhutan

**Keywords:** Wasting, Severe wasting, Children, Child feeding, Bhutan

## Abstract

**Objective:**

To characterize the epidemiology of wasting and identify the main predictors of wasting, severe wasting and poor weight-for-height in children.

**Design:**

We analysed a nationally representative sample of 2028 children (Multiple Indicator Survey, 2010).

**Setting:**

Royal Kingdom of Bhutan.

**Subjects:**

Children aged 0–23 months.

**Results:**

Wasting prevalence was significantly higher among infants aged 0–11 months than among children aged 12–23 months (12·0 *v*. 6·7 %; *P*=0·004) and among boys than girls (11·0 *v*. 7·5 %; *P*=0·04). Children from the Western region had 63 % higher odds of being wasted than children from the Central/Eastern regions (adjusted OR (AOR)=1·63; 95 % CI 1·14, 2·34). Poor feeding practices were among the most significant predictors of wasting and severe wasting. Children who were given prelacteal feeds in the first days of life had 2·5 times higher odds of being severely wasted than those who were not (AOR=2·49; 95 % CI 1·19, 5·19); inadequate complementary feeding in children aged 0–23 months was associated with 58 % higher odds of being wasted (AOR=1·58; 95 % CI 1·02, 2·47) and 2·3 times higher odds of being severely wasted (AOR=2·28; 95 % CI 1·13, 4·58). The association of poor infant feeding practices with wasting and severe wasting was particularly significant in infants (0–11 months).

**Conclusions:**

Programmes for the detection and treatment of severely wasted children need to prioritize very young children (0–11 months), particularly in the Western region. Programmes for the prevention of wasting need to prioritize the improvement of complementary foods and feeding practices in children aged 6–23 months.

Globally, some 50 million children under 5 years of age (under-5s; ~8 % of children aged 0–59 months) suffer from wasting^(^ ^1^ ^)^. Wasting (weight-for-height <−2 sd of the median weight-for-height in the WHO’s Child Growth Standards) poses a serious threat to child survival and development^(^ ^2^ ^)^. Mortality rates in wasted children (weight-for-height *Z*-score (WHZ) <−2) and severely wasted children (WHZ <−3) are three to nine times higher than in children who are not wasted^(^ ^3^ ^)^.

Furthermore, wasted children who survive are at increased risk of stunted growth^(^ ^4^ ^)^. A recent analysis pooling data from eight longitudinal studies in Africa, Asia and Latin America indicated that children with highly variable weight-for-length *Z*-score, negative changes in weight-for-length *Z*-score and/or wasting in the first 17 months of life are at a higher risk of linear growth retardation and stunting at 18–24 months of age, which can result in adverse and often irreversible consequences, including poor cognition and learning performance, reduced lean body mass, short adult stature, lower productivity and reduced earnings^(^ ^5^ ^–^ ^7^ ^)^.

Despite recent economic growth in many countries, South Asia is the global epicentre of child wasting^(^ ^8^ ^)^. The latest data indicate that 16 % of South Asia’s under-5s are wasted. Levels of child wasting in South Asia are almost double those in sub-Saharan Africa (9 %) and four times higher than those in East Asia and the Pacific (4 %)^(^ ^9^ ^)^.

Landlocked at the eastern end of the Himalayas, Bhutan borders China to the north and India to the south, east and west. In 2010, Bhutan’s Multiple Indicator Survey (BMIS) indicated that 6 % of the country’s children aged 0–59 months were wasted and 2 % were severely wasted^(^ ^10^ ^)^. The prevalence of wasting was more than twice higher among children aged 0–23 months than among children aged 24–59 months (9·2 *v*. 3·8 %) while the prevalence of severe wasting was almost four times higher (3·8 *v*. 1·0 %). This is in line with global evidence indicating that most wasting happens during the 1000 d that span from conception until age 2 years^(^ ^11^ ^)^.

The objective of the current analysis was threefold: (i) to characterize the epidemiology of wasting, severe wasting and poor weight-for-height in children aged 0–23 months in Bhutan; (ii) to identify the most significant predictors of wasting, severe wasting and poor weight-for-height in Bhutanese children aged 0–23 months; and (iii) to prioritize areas for action to prevent and treat child wasting in Bhutan.

## Methods

We used publicly available data from the BMIS 2010^(^ ^10^ ^)^. BMIS – the customized version of the Multiple Indicator Cluster Survey (MICS) and the Demographic and Health Survey (DHS) – was a nationally representative household survey designed to provide estimates for indicators on the situation of children and women living in urban and rural areas in the three regions (Central, Eastern and Western) and twenty *dzongkhags* (districts) of the country.

A detailed description of the survey design, sampling methodology, survey tools and data collection can be found elsewhere^(^ ^10^ ^)^. In brief, the urban and rural areas within each *dzongkhag* were the main sampling strata. The sample was selected in two stages; within each stratum, a specified number of blocks in the urban areas and *chiwogs* (municipalities) in the rural areas were randomly selected as enumeration areas with probability proportional to size. Household listing was carried out within the selected enumeration areas and a systematic random sample of households was drawn in each enumeration area.

The survey used three questionnaires: (i) the household questionnaire, administered to collect information on all *de jure* (usual residents) household members, the household and the dwelling; (ii) the women questionnaire, administered in each household to all women aged 15–49 years; and (iii) the children questionnaire, administered to mothers/caregivers of children aged 0–59 months in the household. Once data collection processes were standardized, data collection took place from April to August 2010. Individual consent to participate in the survey was given by the child’s caregiver. The survey included 15 400 households, with a household response rate of 98·4 % and a child response rate of 97·5 %. The survey received ethical clearance from the Research Ethics Board of Health and the National Statistical Bureau.

We selected data from the child data set for children aged 0–23 months and we adjusted for cluster sampling and sampling weights. Wasting and severe wasting were defined as the proportion of children whose WHZ was <−2 or <−3, respectively, using the WHO’s Child Growth Standards^(^ ^2^ ^)^. Analyses were performed using the Stata statistical software package release 12 (2011). In models that performed the regression of wasting or severe wasting as the outcome (dependent) variables *v*. exposure (independent) variables, we report adjusted odds ratios (AOR) and 95 % confidence intervals from logistic regressions. In models that performed the regression of WHZ as the outcome variable *v*. exposure variables, we report regression coefficients and 95 % confidence intervals around point estimates from linear regressions. For all tests, *P* values <0·05 were considered statistically significant. Our analysis did not require ethical approval as we used data that are available for public use and we ensured that data analysis was conducted anonymously.

## Results

The survey included a representative sample of 2404 children aged 0–23 months. Our analytical sample included 2028 children (84·4 %) for whom information on weight and height – and therefore on WHZ, wasting and severe wasting – were available, after eliminating the records of children with missing or implausible anthropometric data (WHZ<−5, WHZ>+5).

### Sample characteristics

The main characteristics of these children are summarized in Table 1: 8·3 % were born with a low birth weight (<2500 g); 33·5 % were born to mothers who were married or entered a marital union before age 18 years; 37·0 % lived in households that used unimproved sanitation facilities; 57·8 % were born to mothers whose delivery was not attended by a doctor, nurse, midwife or skilled personnel; and over 60 % were born to mothers and/or fathers without formal education (62·5 % and 64·5 %, respectively).

**Table 1:** Distribution of children 0–23 months old by socio-economic characteristics. Bhutan, 2010

	Proportion (%)	Number (*n*)
Birth weight		
Weighed at birth	72·0	1380
Not weighed at birth	28·0	536
Birth weight ≥2500 g	91·7	1260
Birth weight<2500 g	8·3	113
Mother’s age		
15–19 years	6·4	127
20–24 years	29·0	576
25–29 years	31·4	623
29–39 years	28·1	558
≥40 years	5·1	101
Parents’ education		
Mother’s education: No formal school education	62·5	1267
Mother’s education: Primary education	14·4	291
Mother’s education: Secondary education	23·2	470
HH head’s education: No formal school education	64·5	1302
HH head’s education: Primary education	14·5	292
HH head’s education: Secondaryeducation	21·0	424
Number of HH members		
≤4	28·7	580
>4	71·3	1438
Water, hygiene and sanitation in the HH		
HH uses improved sources of drinking-water	95·9	1936
HH uses unimproved sources of drinking-water	4·1	82
HH uses an improved sanitation facility	63·0	1272
HH uses an unimproved sanitation facility	37·0	746
HH has a place for handwashing wherewater and soap are available	75·6	1528
HH does not have a place forhandwashing with water and soap available	24·4	494
Mother’s access to health services		
Mother (or her partner) uses acontraceptive method	64·5	1252
Mother (or her partner) does not use acontraceptive method	35·5	687
ANC during last pregnancy provided by adoctor/nurse/midwife	62·7	1173
ANC during last pregnancy provided byother than doctor/nurse/midwife	37·3	697
ANC visits during last pregnancy ≤3	22·6	420
ANC visits during last pregnancy ≥4	77·4	1439
Last delivery was assisted by a doctor/nurse/midwife/skilled staff	42·2	810
Last delivery was not assisted by adoctor/nurse/midwife/skilled staff	57·8	1111
Last delivery took place in a health facility(public or private)	59·8	1149
Last delivery took place at home/other than a health facility	40·2	771

HH, household; ANC antenatal care.

The reported child feeding practices are presented in Table 2. Less than two-thirds (61·2 %) of children were breast-fed within 1 h of birth and less than half (39·9 %) of infants aged 0–5 months were exclusively breast-fed; most children (91·5 %) continued to breast-feed at 1 year, while two-thirds (66·1 %) continued to breast-feed at 2 years. Over two-thirds (67·1 %) of children aged 0–23 months were breast-fed as recommended for their age. About three-quarters (72·0 %) of infants aged 6–8 months were fed complementary foods; a similar proportion (70·6 %) of children 6–23 months old were fed complementary foods a minimum number of times per day (78·4 % in infants 6–11 months old *v*. 67·4 % in children 12–23 months old); and about one in ten children (11·1 %) aged 0–23 months was fed from a bottle with a nipple.

**Table 2:** Breast-feeding and complementary feeding practices in children 0–23 months old. Bhutan, 2010

	Proportion (%)	Number (*n*)
Breast-feeding practices		
Children 0–23 months old breast-fed within 1 h of birth	61·2	1165
Children 0–23 months old breast-fed within 1 d of birth	93·4	1778
Children 0–23 months old who received prelacteal feeds	8·6	176
Children <6 months old who are exclusively breast-fed	39·9	177
Children <6 months old who are predominantly breast-fed	58·5	260
Children 12–15 months old who are breast-fed	91·5	324
Children 20–23 months old who are breast-fed	66·1	222
Children 0–23 months old who are appropriately breast-fed	67·1	1365
Complementary feeding practices		
Children 6–8 months old who are fed CF	72·0	217
Children 6–23 months old who are breast-fed and receive CF	76·1	1206
Children 6–23 months old who receive CF a minimum number of times daily	70·6	957
Children 6–11 months old who receive CF a minimum number of times daily	78·4	309
Children 12–23 months old who receive CF a minimum number of times daily	67·4	648
Children 0–23 months old who are fed from a bottle with a nipple	11·1	224

CF, complementary foods.

### Prevalence of wasting and severe wasting by child, mother and household characteristics

Tables 3–5 summarize the distribution of the three outcome variables – wasting, severe wasting and WHZ – by child (Table 3), maternal (Table 4) and household (Table 5) exposure variables.

**Table 3:** Prevalence of wasting, prevalence of severe wasting and mean weight-for-height *Z*-score (WHZ) in children 0–23 months old by child characteristics. Bhutan, 2010

	Proportion (%) of children wasted (WHZ<−2)	Proportion (%) of children severely wasted (WHZ<−3)	Proportion (%) of wasted children who are severely wasted	Children’s mean WHZ	Number (*n*)
Sex					
Male	11·0	4·1	37·3	−0·16	1047
Female	7·5	2·9	38·7	0·01	981
*P* value	0·04	0·24		0·09	
Age					
0–5 months	14·7	7·4	50·3	−0·17	444
6–11 months	9·7	2·8	28·9	−0·07	522
12–17 months	6·3	2·8	44·4	−0·12	537
18–23 months	7·2	1·3	18·1	0·04	525
*P* value	0·00	0·00		0·04	
Birth weight					
Weighed at birth	9·2	3·6	39·1	−0·01	1380
Not weighed at birth	9·7	3·5	36·1	−0·22	536
*P* value	0·72	0·60		0·02	
Birth weight ≥2500 g	9·2	3·3	35·9	0·03	1260
Birth weight <2500 g	15·4	6·4	41·6	−0·45	113
*P* value	0·09	0·11		0·00	
Breast-feeding practices					
Breast-fed within 1 h of birth	8·8	3·1	35·4	0·00	1165
Not breast-fed within 1 h of birth	10·7	4·2	39·3	−0·16	738
*P* value	0·67	0·49		0·15	
Received prelacteal feeds	10·9	8·1	74·3	−0·30	1852
Did not receive prelacteal feeds	9·1	3·0	33·2	−0·06	176
*P* value	0·83	0·03		0·50	
Is exclusively breast-fed (0–5 months)	18·6	11·6	62·4	−0·38	177
Is not exclusively breast-fed (0–5 months)	11·2	3·8	33·9	0·01	267
*P* value	0·14	0·004		0·14	
Is predominantly breast-fed (0–5 months)	15·9	8·6	54·1	−0·15	260
Is not predominantly breast-fed (0–5 months)	12·3	5·2	42·3	−0·22	184
*P* value	0·60	0·16		0·57	
Is breast-fed (12–15 months)	6·4	3·5	54·7	−0·14	324
Is not breast-fed (12–15 months)	2·9	2·9	100·0	0·67	30
*P* value	0·57	0·77		0·02	
Is breast-fed (20–23 months)	7·4	1·3	17·6	−0·07	222
Is not breast-fed (20–23 months)	10·9	0·0	0·0	0·18	114
*P* value	0·51	0·98		0·38	
Is appropriately breast-fed (0–23 months)	9·2	4·0	43·3	−0·16	1345
Is not appropriately breast-fed (0–23 months)	9·4	2·4	25·2	0·10	683
*P* value	0·33	0·33		0·02	
Complementary feeding practices					
Receives CF (6–8 months)	12·4	4·7	37·9	−0·16	214
Does not receive CF (6–8 months)	9·0	0·0	0·1	0·36	83
*P* value	0·71	0·77		0·27	
Is breast-fed and receives CF (6–23 months)	7·8	2·8	35·5	−0·11	1206
Is not breast-fed and/or does not receive CF (6–23 months)	7·6	0·0	0·1	0·12	378
*P* value	0·88	0·82		0·01	
Receives CF a minimum number of times daily (6–23 months)	9·3	3·1	33·0	−0·13	957
Does not receive CF a minimum number of times daily (6–23 months)	3·7	0·01	0·2	0·01	399
*P* value	0·01	0·18		0·18	
Is fed from a bottle with a nipple (0–23 months)	8·7	3·0	34·9	0·03	224
Is not fed from a bottle with a nipple (0–23 months)	9·3	3·5	37·2	−0·09	1798
*P* value	0·12	0·27		0·15	
Total	9·3	3·5	37·6	0·20	2028

CF, complementary foods.

**Table 4:** Prevalence of wasting, prevalence of severe wasting and mean weight-for-height *Z*-score (WHZ) in children 0–23 months old by maternal characteristics. Bhutan, 2010

	Proportion (%) of children wasted (WHZ<−2)	Proportion (%) of children severely wasted (WHZ<−3)	Proportion (%) of wasted children who are severely wasted	Children’s mean WHZ	Number (*n*)
Mother’s age					
15–19 years	11·3	1·7	15·0	−0·10	127
20–24 years	8·0	4·3	54·0	0·03	576
25–29 years	9·6	3·7	38·4	−0·09	623
29–39 years	10·4	3·5	33·2	−0·19	558
≥40 years	8·9	0·0	0·0	0·13	101
*P* value	0·73	0·27		0·14	
Mother’s education					
None	9·4	3·8	40·4	−0·15	1267
Primary	8·0	2·3	28·8	−0·01	291
Secondary	9·7	3·1	31·6	0·06	470
*P* value	0·77	0·57		0·01	
Mother (or her partner)‘s use of contraception					
Mother (or her partner) uses a contraceptive method	9·1	3·0	33·0	−0·07	1252
Mother (or her partner) does not use a contraceptive method	10·2	4·7	46·1	−0·08	687
*P* value	0·61	0·14		0·60	
Mother’s use of ANC					
ANC was provided by a doctor/nurse/midwife	7·2	2·0	27·6	−0·10	1173
ANC was provided by other than a doctor/nurse/ midwife	10·3	4·4	42·2	0·01	697
*P* value	0·07	0·01		0·33	
Number of ANC visits during the last pregnancy ≤3	9·2	4·6	50·0	−0·15	420
Number of ANC visits during the last pregnancy ≥4	9·3	3·3	35·5	−0·04	1439
*P* value	0·38	0·66			0·70
Mother’s assistance at/place of delivery					
Mother’s last delivery was assisted by a doctor/ nurse/midwife	8·4	3·3	39·3	0·00	810
Mother’s last delivery was not assisted by a doctor/ nurse/midwife	10·3	3·7	35·9	−0·11	1111
*P* value	0·16	0·35		0·56	
Mother’s place of delivery					
Mother delivered last child in a health facility (public or private)	9·4	3·5	37·2	−0·02	1149
Mother delivered last child at home/not in a facility (public or private)	9·7	3·6	37·1	−0·14	771
*P* value	0·38	0·31		0·09	
Under-age marriage					
Mother was married or entered a marital union before age 18 years	8·8	3·3	37·5	−0·07	659
Mother was married or entered marital union at age ≥18 years	9·8	3·7	37·8	−0·08	1306
*P* value	0·51	0·60		0·31	
Domestic violence					
Mother believes husband is justified in beating his wife/partner	8·6	2·9	33·7	−0·07	1415
Mother believes husband is not justified in beating his wife/partner	11·1	5·2	46·4	−0·08	525
*P* value	0·21	0·27		0·90	
Total	9·3	3·5	37·6	0·20	2028

ANC antenatal care.

**Table 5:** Prevalence of wasting, prevalence of severe wasting and mean weight-for-height *Z*-score (WHZ) in children 0–23 months old by household characteristics. Bhutan, 2010

	Proportion (%) of children wasted (WHZ<−2)	Proportion (%) of children severely wasted (WHZ<−3)	Proportion (%) of wasted children who are severely wasted	Children’s mean WHZ	Number (*n*)
Residence					
Urban	11·1	4·7	42·3	0·01	456
Rural	8·5	2·9	34·1	−0·12	1572
*P* value	0·05	0·3		0·31	
Region					
Western	12·8	4·9	38·3	−0·24	661
Central	6·1	2·9	47·5	−0·02	826
Eastern	6·4	1·6	25·0	0·13	541
*P* value	0·002	0·07		0·01	
Wealth index					
Poorest	7·1	1·9	26·8	−0·14	404
Second	11·8	3·0	25·4	−0·21	435
Middle	7·9	4·2	53·2	0·06	429
Fourth	8·9	2·8	31·5	−0·04	400
Richest	11·3	5·7	50·4	−0·05	317
*P* value	0·63	0·12		0·00	
HH head’s education					
None	8·2	2·7	32·9	−0·04	1302
Primary	8·7	4·3	49·4	−0·22	292
Secondary	12·4	4·8	38·7	−0·09	424
*P* value	0·08	0·05		0·26	
Number of HH members					
≤4	9·0	3·3	36·7	−0·01	580
>4	9·4	3·5	37·2	−0·11	1438
*P* value	0·85	0·88		0·21	
Water, hygiene and sanitation in HH					
HH uses improved sources of drinking-water	9·0	3·2	35·6	−0·06	1936
HH uses unimproved sources of drinking-water	15·9	10·1	63·5	−0·44	82
*P* value	0·44	0·10		0·43	
HH uses improved sanitation facilities	9·6	3·9	40·6	−0·08	1272
HH uses unimproved sanitation facilities	8·7	2·5	28·7	−0·07	746
*P* value	0·54	0·14		0·15	
HH has a place for handwashing where water and soap are available	9·8	3·3	33·7	−0·08	1528
HH does not have a place for handwashing with water and soap available	6·1	3·9	63·9	−0·04	494
*P* value	0·36	0·35		0·60	2022
Total	9·3	3·5	37·6	0·20	2028

HH, household.

Table 3 (child characteristics) indicates that the prevalence of wasting in children 0–23 months old was 9·3 % while the prevalence of severe wasting was 3·5 %: thus over one-third (37·6 %) of the wasted children were severely wasted. The proportion of wasted children declined with age (from 14·7 % in children aged 0–5 months to 7·2 % in children aged 18–23 months; *P*<0·005), as did the proportion of severely wasted children (from 7·4 % in children aged 0–5 months to 1·3 % in children aged 18–23 months; *P*=0·003). The mean WHZ improved with age (from −0·17 in children aged 0–5 months to +0·04 in children aged 18–23 months; *P*=0·04).

The prevalence of wasting was significantly higher in boys than in girls (11·0 *v*. 7·5 %; *P*=0·04) and in children 0–11 months old than in children 12–23 months old (12·0 *v*. 6·7 %, *P*=0·004; data not presented). The prevalence of severe wasting was significantly higher among children 0–11 months old than among children 12–23 months old (5·0 *v*. 2·1 %, *P*=0·009; data not presented), in infants who were fed prelacteal feeds in the first days of life (8·1 *v*. 3·0 %; *P*=0·03) and in children aged 0–5 months who were exclusively breast-fed (11·6 *v*. 3·8 %; *P*=0·004). The mean WHZ was significantly poorer among children who were not weighed at birth (*P*=0·02), children born with a low weight (<2500 g; *P*<0·001), children 0–11 months old (*P*=0·04), and children 0–23 months old who were breast-fed as recommended for their age (*P*=0·02).

Table 4 (maternal characteristics) indicates that the prevalence of severe wasting was significantly higher among children whose mothers received prenatal care by other than a doctor/nurse/midwife (4·4 *v*. 2·0 %; *P*=0·01). The mean WHZ was significantly poorer among children born to mothers without any formal education (*P*=0·01) and children whose mothers were not married/in union at the time of the survey (*P*=0·05; data not presented).

Table 5 (household characteristics) indicates that the prevalence of wasting and severe wasting were significantly higher (i.e. double) in the Western region than in the Central and Eastern regions (12·8 *v*. 6·1 and 6·4%, *P*=0·002 and 4·9 *v*. 2·9 and 1·6%, *P*=0·07 for wasting and severe wasting in the Western, Central and Eastern region, respectively). The prevalence of wasting tended to be significantly higher in urban than in rural areas (11·1 *v*. 8·5 %; *P*=0·05). The mean WHZ was significantly poorer in children from the Western region (*P*=0·01) and children from the two lowest wealth quintiles (*P*<0·01).

### Multivariate regression analysis: main predictors of child wasting in Bhutan

Multivariate logistic regression – after adjusting for age, sex, residence and selected caregiver and household-level variables – indicated that three variables were independently associated with wasting in children 0–23 months old: age, region and complementary feeding (Table 6). Children aged 0–11 months had a 46 % higher odds of being wasted than children aged 12–23 months (AOR=1·46; 95 % CI 1·00, 2·13). Children aged 0–23 months from the Western region had 63 % higher odds of being wasted than those from the Central and Eastern regions (AOR=1·63; 95 % CI 1·14, 2·34), while children aged 0–11 months from the Western region had a 2·2-fold higher odds of being wasted than those from the Central/Eastern regions (AOR=2·24; 95 % CI 1·39, 3·60). Finally, the odds of being wasted were 58 % higher in children 0–23 months old who were not fed complementary foods as appropriate for their age (AOR=1·58; 95 % CI 1·02, 2·47); this association was even stronger in children aged 0–11 months as the odds of being wasted were about twofold higher in children aged 0–11 months old who were not fed complementary foods as appropriate for their age (AOR=1·96; 95 % CI 1·21, 3·16; Table 6).

**Table 6:** Adjusted odds ratios (AOR) of wasting and severe wasting by age group in relation to child, maternal and household characteristics. Bhutan, 2010

	Wasting						Severe wasting					
	0–11 months		12–23 months		0–23 months		0–11 months		12–23 months		0–23 months	
	AOR	95 % CI	AOR	95 % CI	AOR	95 % CI	AOR	95 % CI	AOR	95 % CI	AOR	95 % CI
Age												
12–23 months	1·0	Ref.	1·0	Ref.	1·0	Ref.	1·0	Ref.	1·0	Ref.	1·0	Ref.
0–11 months	n/a	n/a	n/a	n/a	1·46	1·00, 2·13	n/a	n/a	n/a	n/a	1·59	0·87, 2·91
Sex												
Female	1·0	Ref.	1·0	Ref.	1·0	Ref.	1·0	Ref.	1·0	Ref.	1·0	Ref.
Male	1·23	0·78, 1·96	1·52	0·87, 2·67	1·35	0·95, 1·92	1·34	0·66, 2·68	1·03	0·43, 2·49	1·20	0·70, 2·05
Residence												
Urban	1·0	Ref.	1·0	Ref.	1·0	Ref.	1·0	Ref.	1·0	Ref.	1·0	Ref.
Rural	0·84	0·47, 1·50	0·76	0·39, 1·47	0·79	0·52, 1·20	1·32	0·56, 3·14	1·46	0·47, 4·59	1·30	0·65, 2·59
Region												
Central/Eastern	1·0	Ref.	1·0	Ref.	1·0	Ref.	1·0	Ref.	1·0	Ref.	1·0	Ref.
Western	2·24	1·39, 3·60	1·05	0·57, 1·92	1·63	1·14, 2·34	1·51	0·74, 3·08	0·86	0·34, 2·17	1·25	0·72, 2·18
HH head’s education												
Secondary	1·0	Ref.	1·0	Ref.	1·0	Ref.	1·0	Ref.	1·0	Ref.	1·0	Ref.
None	1·03	0·58, 1·83	0·75	0·38, 1·49	0·91	0·59, 1·40	0·99	0·43, 2·29	0·43	0·15, 1·20	0·72	0·36, 1·43
Who provides ANC												
ANC provided by a doctor/nurse/midwife	1·0	Ref.	1·0	Ref.	1·0	Ref.	1·0	Ref.	1·0	Ref.	1·0	Ref.
ANC provided by other than doctor/nurse/midwife	1·22	0·72, 2·08	1·08	0·58, 2·01	1·18	0·66, 2·11	2·31	1·01, 5·36	2·05	0·63, 6·66	2·21	1·13, 4·33
Prelacteal feeding (0–23 months)												
Did not receive prelacteal feeds	1·0	Ref.	1·0	Ref.	1·0	Ref.	1·0	Ref.	1·0	Ref.	1·0	Ref.
Received prelacteal feeds	1·49	0·68, 3·25	0·86	0·34, 2·17	1·22	0·68, 2·18	4·33	1·76, 10·66	0·93	0·21, 4·10	2·49	1·19, 5·19
Age-appropriate breast-feeding												
Children are not breast-fed as appropriate for their age	1·0	Ref.	1·0	Ref.	1·0	Ref.	1·0	Ref.	1·0	Ref.	1·0	Ref.
Children are breast-fed as appropriate for their age	1·07	0·65, 1·78	0·64	0·32, 1·26	0·98	0·64, 1·19	2·51	1·13, 5·51	0·62	0·21, 1·79	1·79	0·87, 3·69
Age-appropriate complementary feeding												
Child is fed CF as appropriate for age	1·0	Ref.	1·0	Ref.	1·0	Ref.	1·0	Ref.	1·0	Ref.	1·0	Ref.
Child is not fed CF as appropriate for age	1·96	1·21, 3·16	0·59	0·18, 1·88	1·58	1·02, 2·47	2·94	1·42, 6·08	0·42	0·05, 3·70	2·28	1·13, 4·58

HH, household; ANC, antenatal care; CF, complementary foods; ref., reference category.

Four variables were independently associated with severe wasting in children 0–23 months old: antenatal care, prelacteal feeding, breast-feeding and complementary feeding. The odds of being severely wasted were 2·2-fold higher in children aged 0–23 months whose mothers did not receive antenatal care by a doctor, nurse or midwife (AOR=2·21; 95 % CI 1·13, 4·33); similarly, the odds of being severely wasted were 2·5-fold higher in children who were fed prelacteal feeds (AOR=2·49; 95 % CI 1·19, 5·19) and 2·3-fold higher among children who were not fed complementary foods as appropriate for their age (AOR=2·28; 95 % CI 1·13, 4·58). The association of infant feeding variables with severe wasting was particularly strong in children 0–11 months old; the odds of being severely wasted were fourfold higher in children 0–11 months old who were given prelacteal feeds (AOR=4·33; 95 % CI 1·76, 10·66) and threefold higher among those who were not fed complementary foods as appropriate for their age (AOR=2·94; 95 % CI 1·42, 6·08). Breast-feeding was associated with a higher odds of severe wasting in infants 0–11 months old (AOR=2·51; 95 % CI 1·13, 5·51; Table 6).

The models performing the regression of WHZ on the exposure variables indicated that the likelihood of poor WHZ was significantly higher among children who were not weighed at birth (*P*<0·05), children of mothers without formal education (*P*<0·01) and children from the Western region (*P*<0·01; Table 7).

**Table 7:** Associations between exposure variables and child weight-for-height *Z*-score (WHZ) in children 0–23 months old. Bhutan, 2010

	Dependent variable: WHZ (0–23 months old)	
Independent variable	Coefficient	95 % CI
Age		
Child’s age was 0–11 months *v*. Child’s age was 12–23 months	−0·023	−0·170, 0·120
Weighed at birth		
Child was weighed at birth *v*. Child was not weighed at birth	0·187*	0·310, 0·340
Age-appropriate complementary feeding (0–23)		
Child was not fed CF when appropriate for age *v*. Child was fed CF when appropriate for age	0·155	−0·040, 0·358
Mother’s education		
Child’s mother had no formal education *v*. Child’s mother completed primary or secondary education	0·239*	−0·084, 0·393
Wealth index		
Child belonged to bottom 50 % poorer households *v*. Child belonged to top 50 % wealthier households	−0·047	−0·201, 0·107
Region		
Child lived in the Western region *v*. Child lived in the Central/Eastern region	−0·230**	−0·381, −0·079

CF, complementary foods. *P<0·05, ** *P*<0·01.

## Discussion

Between 1988 and 2010, the prevalence of wasting in Bhutanese children aged 0–59 months hovered around 6 % without much improvement, with levels of wasting and severe wasting two to four times higher among children aged 0–23 months than among children aged 24–59 months^(^ ^10^ ^,^ ^12^ ^)^. We used data from BMIS 2010 to characterize the epidemiology of wasting in children 0–23 months old in Bhutan, identify the most significant predictors of wasting, severe wasting and poor weight-for-height, and – on the basis of these findings – prioritize areas for action.

We found that almost one in ten children (9·3 %) aged 0–23 months was wasted and over one-third (37·6 %) of the wasted children were severely wasted. The prevalence of wasting was almost double among infants 0–11 months old than among children aged 12–23 months while the prevalence of severe wasting was 2·4-fold higher among infants 0–11 months old than among children aged 12–23 months, suggesting that most wasting happens either prenatally or in the first year of life. It has been documented that, compared with the WHO/National Center for Health Statistics 1976 reference, use of the WHO 2006 Child Growth Standards changes the age pattern of wasting in children of pre-school age, with significantly increased levels of wasting in early and late infancy (0–5 months and 6–11 months, respectively) than in early childhood (12–23 months)^(^ ^13^ ^)^.

Some of the most relevant findings of our analysis are related to the significant association of infant feeding practices with wasting, severe wasting and attained WHZ. Poor complementary feeding practices – not aligned with internationally agreed-upon guidance – were the variables more systematically associated with wasting and severe wasting in children aged 0–23 months. Global and national policy recommends that infants aged 0–5 months be exclusively breast-fed, with no other fluids or foods given, not even water, while children aged 6–23 months should be fed age-appropriate soft, semi-solid or solid complementary foods while breast-feeding continues^(^ ^14^ ^)^. In our sample, 28 % of infants aged 6–8 months were not being fed complementary foods (timely introduction of complementary feeding), 23·9 % of children aged 6–23 months were not being fed both breast milk and complementary foods (complementary feeding with continued breast-feeding) and 29·4 % of children aged 6–23 months were not being fed complementary foods a minimum number of times per day (minimum meal frequency). Children 0–23 months old who were not fed complementary foods as recommended for their age had 1·6-fold higher odds of being wasted and 2·3-fold higher odds of being severely wasted after controlling for all other variables. This association was even stronger among infants aged 0–11 months: those who were not fed complementary foods as recommended for their age had twofold higher odds of being wasted and threefold higher odds of being severely wasted. A recent multi-country analysis of DHS data in eight countries found that several indicators of appropriate complementary feeding were positively associated with higher mean WHZ and/or lower odds of wasting in Uganda, Zambia and Zimbabwe^(^ ^15^ ^)^.

Prelacteal feeding (i.e. non-exclusive breast-feeding in the first 3 d of life) was associated with 2·5-fold higher odds of severe wasting in children 0–23 months old and 4·3-fold higher odds of severe wasting in infants 0–11 months old. However, age-appropriate breast-feeding (exclusive breast-feeding in infants aged 0–5 months and continued breast-feeding in children aged 6–11 months) was associated with 2·5-fold higher odds of severe wasting in infants aged 0–11 months (AOR=2·51; 95 % CI 1·13, 5·51). This finding is counter-intuitive and deserves further investigation. Studies in Bangladesh and Zambia found a positive association between exclusive breast-feeding and reduced odds of wasting^(^ ^16^ ^,^ ^17^ ^)^. Conversely, studies in Ethiopia, Haiti, India, Kenya, Uganda and Zimbabwe did not find any significant positive association between breast-feeding and lower odds of wasting or higher attained WHZ^(^ ^15^ ^,^ ^17^ ^–^ ^19^ ^)^. A recent meta-analysis on the growth benefits of breast-feeding has shown that breast-feeding interventions were associated with small, non-significant increases in weight and length/height *Z*-scores, and led to a modest, albeit significant, reduction in BMI *Z*-score/WHZ. For all outcomes, there was substantial heterogeneity among studies, which led the authors of the meta-analysis to indicate that their results ‘must be interpreted with caution’^(^ ^20^ ^)^.

However, the benefits of breast-feeding for child survival and development are well established^(^ ^21^ ^,^ ^22^ ^)^. Recent meta-analyses and systematic literature reviews by WHO show that children who are breast-fed have better survival rates, higher intelligence quotients, and lower risk of otitis, malocclusion, asthma and obesity. Breast-feeding mothers benefit from having breast-fed, with lower rates of breast cancer, ovarian cancer, type 2 diabetes and postpartum depression. The multiple benefits of breast-feeding demonstrate the contribution and relevance of breast-feeding as a global public health issue, for low- and high-income populations alike^(^ ^23^ ^)^. Therefore the protection, promotion and support of optimal breast-feeding needs to remain a central component of national programmes for child survival and development even if the empirical evidence of its direct impact on the prevention of wasting is limited^(^ ^14^ ^,^ ^24^ ^)^.

On the basis of our key findings we identify three policy and programme priorities:

1.
Programmes for the detection and treatment of severely wasted children need to prioritize very young children (0–11 months), particularly in the Western region. Bhutan should consider adopting the community management of acute malnutrition (CMAM) approach for the early detection and care of children with severe wasting. Over fifty countries have adopted this approach^(^ ^25^ ^,^ ^26^ ^)^. CMAM should be scaled up as part of a continuum of care for the prevention and treatment of undernutrition in infancy and early childhood.
2.
Programmes for the prevention of child wasting need to prioritize the improvement of complementary foods and feeding practices. There is agreement on the population-based indicators – timely introduction, feeding frequency, diet diversity, safe and responsive feeding – that should inform programme design and monitoring^(^ ^27^ ^,^ ^28^ ^)^. A nutrition surveillance system that provides information on these indicators – many of which were not included in BMIS 2010 – as well as information on seasonal and geographical variations in household food insecurity and child growth should inform a nationwide effort to improve children’s access to age-appropriate nutritious complementary foods.
3.
Programmes for the prevention of wasting need to ensure that women have access to information and support. While national efforts on girls’ education are scaled up, attention needs to be provided to mothers with no/less formal education through supportive interventions like antenatal care. Our analysis shows that mothers’ access to antenatal care was associated with a significantly lower risk of severe wasting in children, while being weighed at birth (a proxy for access to skilled delivery) was positively associated with WHZ in children. Antenatal and perinatal care provide an important platform to ensure that pregnant women receive counselling and support to improve the diets of their children in the first months of life.

Previous analyses have shown that better antenatal and perinatal care for women during pregnancy and delivery and appropriate complementary feeding for children in the first 2 years of life were also protective against child stunting in Bhutan^(^ ^29^ ^)^.

## Conclusion

We analysed a nationally representative sample of 2028 children aged 0–23 months to characterize the epidemiology of wasting in Bhutan. We found that the prevalence of wasting was significantly higher among infants aged 0–11 months and children from the Western region. Poor feeding practices were among the most significant predictors of wasting and severe wasting, particularly among infants 0–11 months old. Prelacteal feeding in the first days of life and late introduction of complementary foods more than doubled the odds of severe wasting. Programmes for the prevention of child wasting in Bhutan need to prioritize the improvement of complementary foods and feeding practices in children aged 6–23 months, while programmes for the detection and treatment of severely wasted children need to prioritize very young children (0–11 months), particularly in the Western region.
